# Effective Management of Femur Fracture Using Damage Control Orthopedics Following Fat Embolism Syndrome

**DOI:** 10.7759/cureus.7455

**Published:** 2020-03-29

**Authors:** Abralena Wilson, Adel Hanandeh, Ahmed A Shamia, Kevin Louie, Brian Donaldson

**Affiliations:** 1 Surgery, Columbia University College of Physicians and Surgeons at Harlem Hospital Center, New York, USA; 2 General Surgery, Columbia University College of Physicians and Surgeons at Harlem Hospital Center, New York, USA; 3 Surgery, Harlem Hospital Center, New York, USA; 4 General Surgery, St. Barnabas Hospital Health System, Bronx, USA

**Keywords:** fat embolism, damage control orthopedic, femur fracture, mental status, petechia

## Abstract

Fat embolism syndrome (FES) is a rare event following a traumatic injury, and its pathophysiologic mechanism continues to be elusive. Fat embolism syndrome generally occurs when a bone marrow fat enters the bloodstream resulting in a cascade of inflammatory response, hyper-coagulation, and an array of symptoms that generally begin within 24-48 hours. FES early symptoms include petechial rash, shortness of breath, altered mental status, seizures, fever, and may result in decreased urine output. The common etiologies of a fat embolism include long bone fractures, mainly femoral and pelvic fractures.

There are multiple management methods described in the literature to help prevent FES and other long bone fracture complications from occurring. Although not universally adopted, the damage control orthopedics (DCO) has been the major management option for patients with a long bone fracture. DCO is entertained by provisional immobilization of patients with long bone fractures and those who are considered severely traumatized patients (STP). Thus, immobilization can help minimize the traumatic effect and the subsequent second hit by performing non-life saving surgical procedures.

In this case, a patient with a transverse femur fracture suffered disconcerting symptoms of fat embolism prior to definitive femur repair. Hence, damage control orthopedics was entertained with a postponement of his femur repair to facilitate stabilization. The use of damage control orthopedics was successful in this patient with no long term complications.

## Introduction

Damage control orthopedics (DCO) became popular during World War I. Wounded soldiers with long bone fractures-especially femoral fractures-had devastating outcomes, with mortality rates reaching up to 80 percent. The introduction of Thomas splint (femur bone splint) during World War I made a great impact by decreasing the mortality rate down to 20 percent. The use of Thomas splint, and, by proxy, applying DCO, helped stabilize patients prior to any definitive surgical interventions. Damage control orthopedics allowed for enough time to medically optimize those in need prior to more invasive interventions.

This method has been increasingly adopted worldwide, but data is limited with respect to risk, cost, and long-term outcomes. Few studies have shown that the outcome of damage control orthopedics mainly depends on the initial degree of injury, the body's biological response, and the type of treatment required [1-3]. Current literature also proposes that DCO utilization would promote temporary fracture stabilization, decreased blood loss, decreased secondary inflammatory insult due to definitive repair, and, finally, better pain control [1-3]. Therefore, the prevention of superimposed secondary hit inflammatory response would reduce the chance of worsening coagulopathies, fat embolism, infection, and poor wound healing.

In this article, we present a case report of a trauma patient with a long bone fracture that was complicated by fat embolism syndrome. This case was successfully managed by damage control orthopedics before definitive repair, allowing enough time for the inflammatory response to subside, decreasing morbidity, and promoting rapid recovery. 

## Case presentation

**Figure 1:**
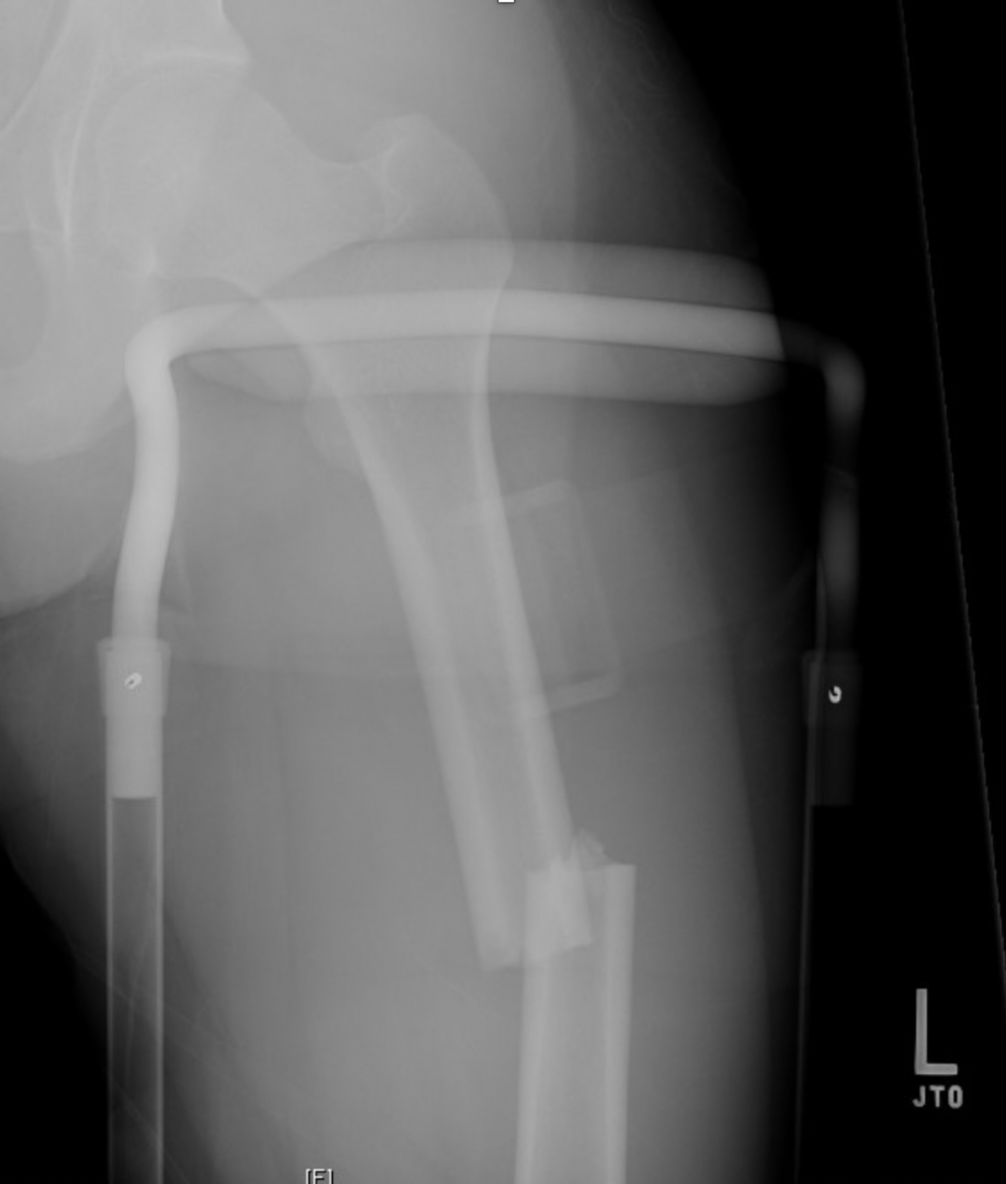
Initial traction of left extremity prior to definitive repair

A 23-year-old male pedestrian was struck and presented as a level 2 trauma. On admission, the patient was hemodynamically stable, afebrile, with a Glasgow coma scale (GCS) of 15. On physical examination, the left lower extremity was found to be shortened and externally rotated. The patient had no other injuries or fractures on primary and secondary surveys, respectively. Left-thigh x-rays indicated an isolated transverse left femur fracture in the proximal and mid femoral diaphysis with lateral displacement of the distal fracture. The patient was admitted to the trauma service, and traction was applied to his left lower extremity with a planned definitive repair of femur fracture (Figure 1).

On hospital day two, the patient developed pyrexia, tachycardia, tachypnea, altered mental status, with petechial rashes on his upper extremities and axilla bilaterally. The surgical procedure was deferred, and supportive care was entertained.

**Figure 2:**
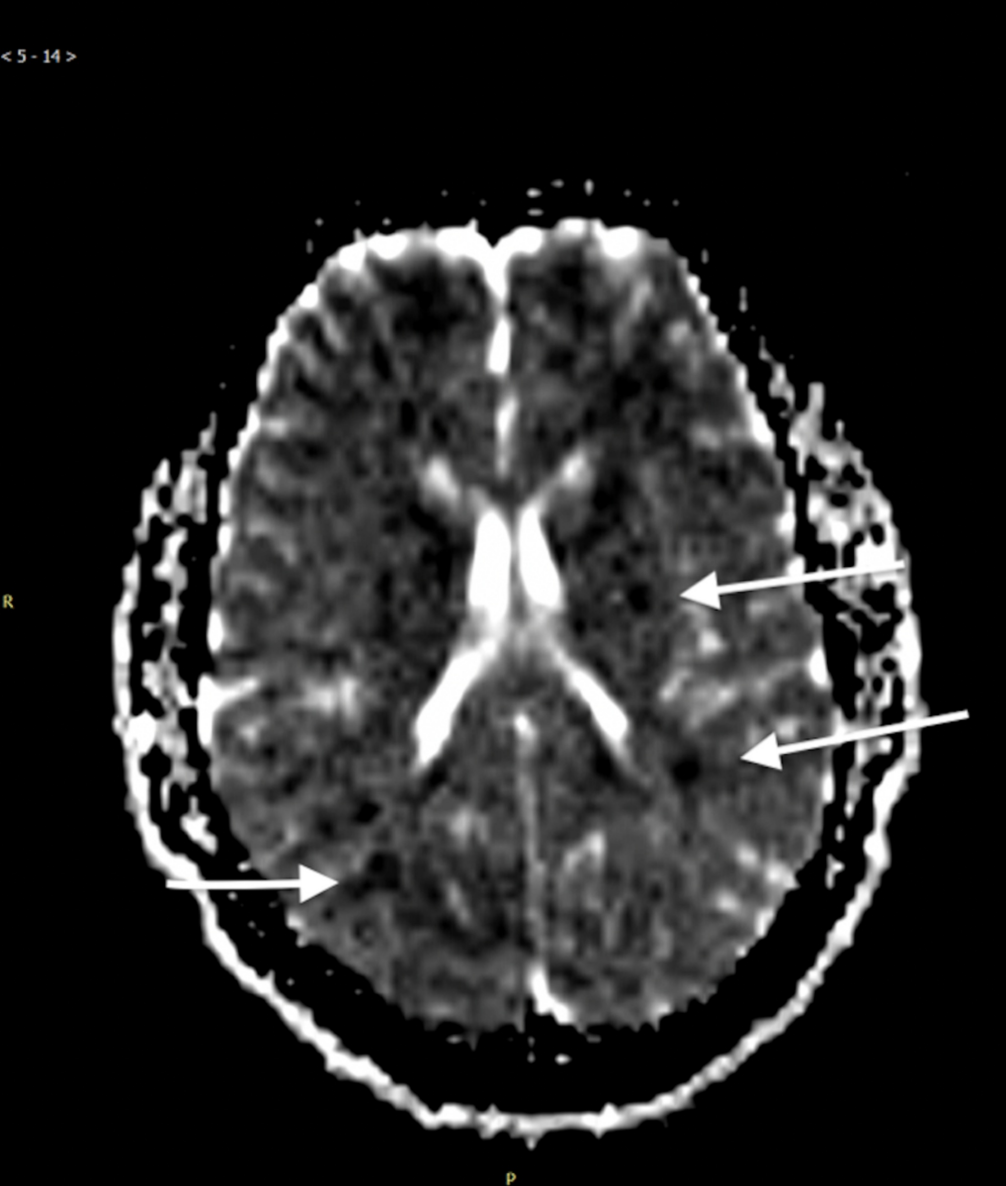
MRI of the brain indicated evidence of scattered embolic ischemia secondary to cerebral fat embolism

Subsequently, the patient was found to have a decrease in his GCS to 7 (eye response (E):1, verbal response (V):1, motor response (M):5), and he was found to be in acute respiratory failure requiring mechanical ventilatory support. A chest x-ray was significant only for a left upper lobe infiltrate. Chest computed tomography angiography was negative for pulmonary embolism, and non-contrast CT head was negative for any intracranial pathologies. However, brain magnetic resonance imaging (MRI) indicated evidence of scattered embolic ischemia or starfield pattern, which was diagnostic for cerebral fat embolism or fat embolism syndrome (Figure 2). 

**Figure 3:**
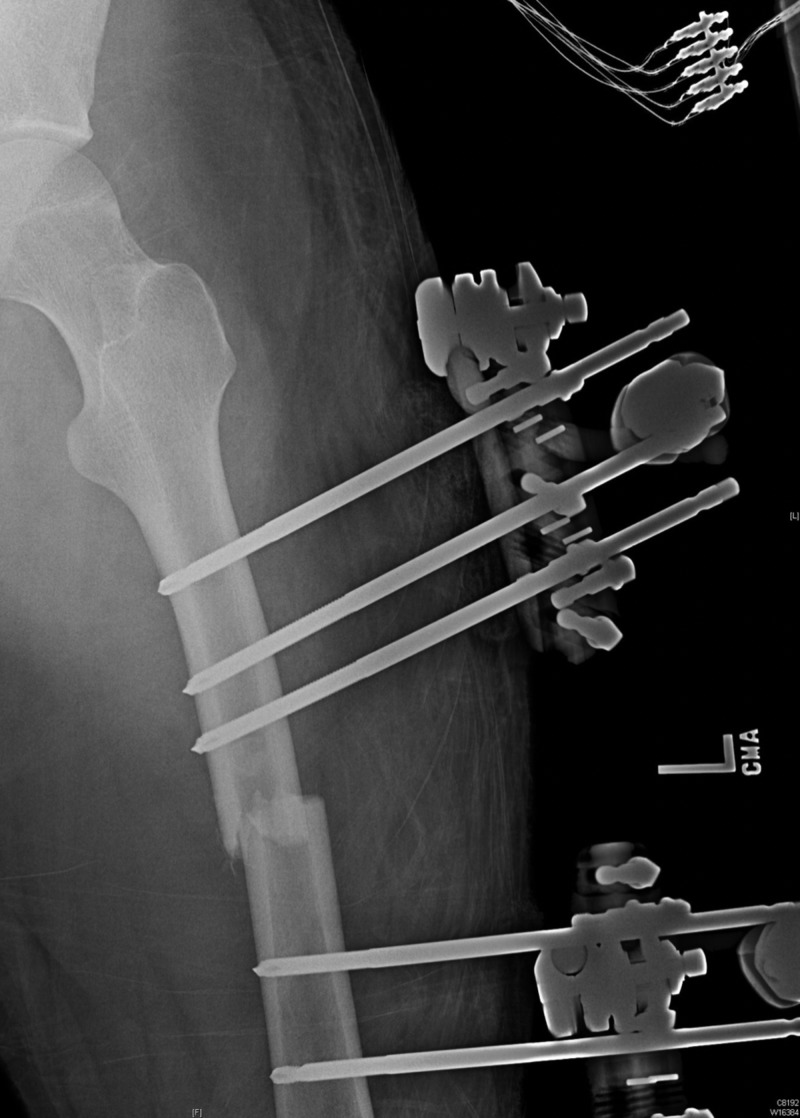
External fixation of the left extremity

**Figure 4:**
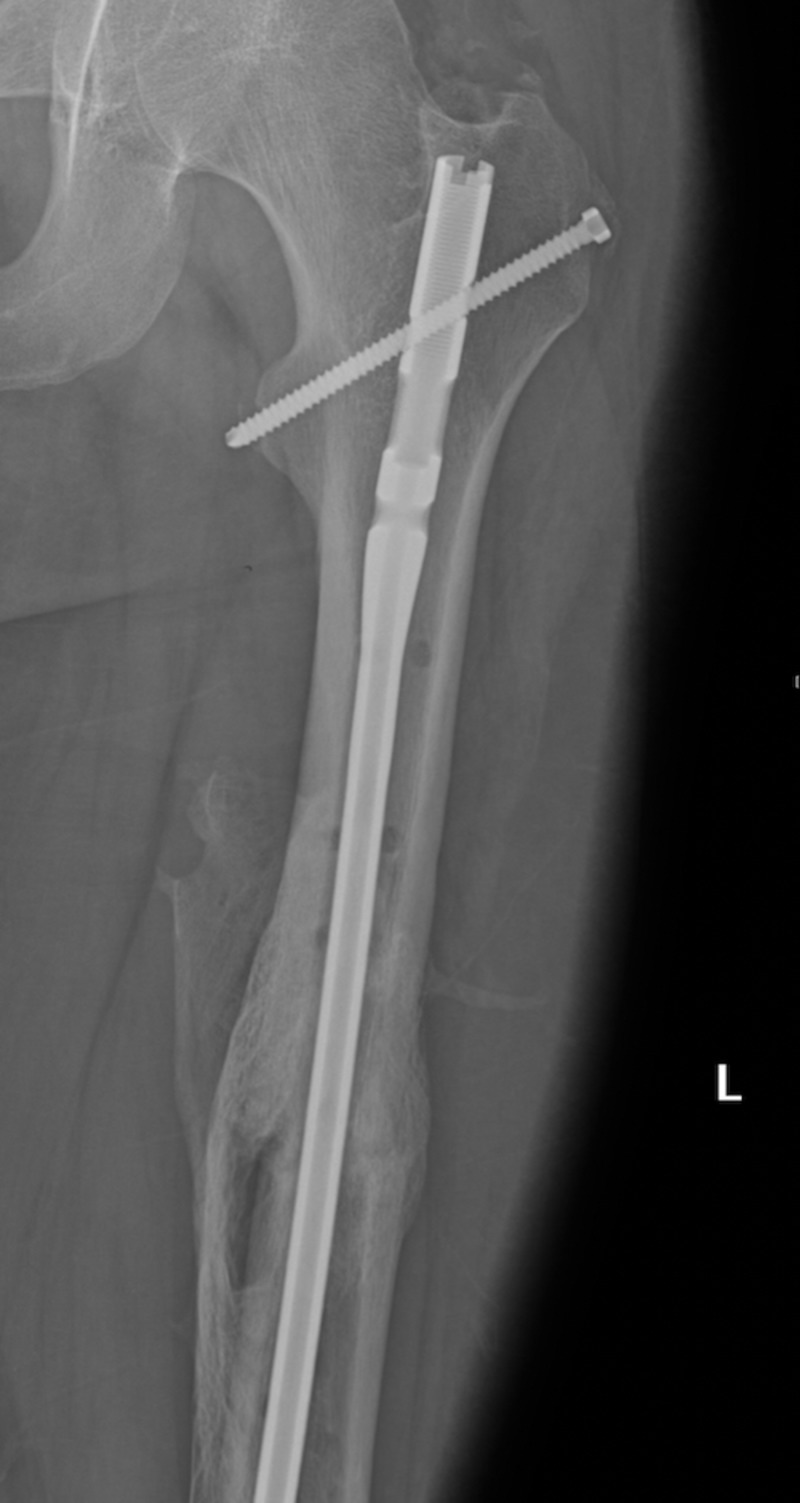
Definitive fracture stabilization with intramedullary rodding fixation

On hospital day seven, the patient underwent temporary stabilization by external fixation (Figure 3). On hospital day twenty-two, the patient underwent definitive repair with open reduction, internal fixation, and intramedullary rod placement (Figure 4). The patient did not have any postoperative complications, and his mental status was back to normal. Subsequently, the patient was discharged to a subacute rehabilitation center on hospital day twenty-nine. 

Four weeks later, the patient returned for an outpatient clinic follow up. He was found to have recovered most of his motor strength and was able to ambulate with no restriction. 

## Discussion

Fat embolism syndrome (FES) is defined by the presence of fat globules in pulmonary or cerebral microcirculation, which may or may not show any clinical signs. In current literature, there are two main theories on the pathophysiology of FES: mechanical and biochemical. The mechanical theory proposes that potent inflammatory/prothrombotic factors such as the bone marrow fat cells access the circulatory system, leading to a hyper-thrombotic state. This triggers platelet aggregation and the creation of micro- or macro-thrombi that travel to the pulmonary arterial circulation or bypass the pulmonary circulation and access the systemic circulation via a patent foramen ovale leading to what's currently known as fat embolism syndrome [1,4-6].

The biochemical theory presumes that FES is due to the inflammatory changes in the bone marrow itself. As bone marrow fat globules are broken down by tissue lipase, the byproducts such as glycerol and fatty acids would leak into the circulatory system leading to multi-organ failure. This inflammatory insult results in organ dysfunction such as injury to the pulmonary pneumocytes and vascular endothelial cells, leading to an array of vasogenic edema, cytotoxic edema, and hemorrhage. Ultimately, a vicious cycle ensues by the continuous release of pro-inflammatory cytokines by damaged endothelial cells leading to fat embolism syndrome (FES) in non-traumatic settings with no bone fractures. 

While there is no universal consensus on the definition of FES, many scholars have proposed diagnostic criteria. Most commonly used are the Gurd's and Wilson's criteria, which are categorized into major features and minor features. The major criteria include petechial rash, hypoxia, and central nervous system (CNS) changes. The minor criteria include tachycardia, pyrexia, anemia, thrombocytopenia, high ESR, lipuria, and sputum fat. A diagnosis of FES can be made if one major feature plus four minor features with the addition of fat macroglobulinemia are present. It's important to note that most recent literature has removed the presence of fat macroglobulinemia as a requirement for an FES diagnosis [2,6,7].

The most important measures that have been shown to decrease the incidence of FES are the fewer number of bone fractures and the lower degree of fracture displacement. Those who require DCO are patients with more extensive fractures and higher baseline risk of fat emboli formation. Thus, early fracture stabilization with delayed definitive repair was most safe and effective [2,3,8,9].

The timing of surgery has important implications in the overall inflammatory response. For example, Tuttle and colleagues indicated that DCO was a safe initial treatment for trauma patients with multiple injures, including those with femur shaft fractures [10]. They concluded there were a few disadvantages to the use of damage control orthopedics: higher medical costs, longer hospital stay, and greater occurrences of deep vein thrombosis and nosocomial infections [2,9-11].

In this case, there were two major Gurd's criteria noted: petechial rash and CNS changes. Also, four minor criteria were noted, including tachycardia, pyrexia, a sudden drop of hemoglobin, and acute thrombocytopenia. In our patient, perioperative fat emboli syndrome was diagnosed secondary to the first hit injury/femur fracture. A decision was made to apply damage control orthopedic with stabilization and external fixation to allow the inflammatory process to subside until a more definitive repair was possible.

The purpose of this case is to shine a light on damage control orthopedics and its utilization prior to definitive surgical repair in patients with FES syndrome to decrease any long-term complications. Finally, current orthopedic and trauma literature shows the utilization of damage control orthopedic prior to definite repair, particularly for polytrauma patients or high-risk patients with multiple co-morbidities such as adult respiratory distress syndrome and end-organ failure [2,3,8-11].

## Conclusions

Our patient sustained perioperative fat embolism and had a full recovery of all neurological symptoms after applying damage control orthopedics prior to secondary definitive fracture repair. It’s important for trauma surgeons to aware of the indications and benefits of damage control orthopedics, especially for polytrauma patients with multiple long bone fractures. Current literature strongly encourages fracture stabilization by a temporary fixation to help control and subside the first hit inflammatory responses, initial injury or fracture. Entertaining damage control orthopedics in patients with fat embolism after the first-hit/bone-fracture is the best option to help control the inflammatory insult and prevent against FES formation. Finally, damage control orthopedics is an optimal choice after FES to help subside the inflammatory response and prevent the formation of more emboli, thus, halting the cerebral or pulmonary insults.
